# Towards PPG-based anger detection for emotion regulation

**DOI:** 10.1186/s12984-023-01217-5

**Published:** 2023-08-15

**Authors:** Tuck-Voon How, Robin E. A. Green, Alex Mihailidis

**Affiliations:** 1 Institute of Biomedical Engineering, University of Toronto, Toronto, ON Canada; 2 KITE Research Institute, Toronto Rehabilitation Institute-University Health Network, Toronto, ON Canada; 3 Department of Psychiatry, University of Toronto, Toronto, ON Canada

**Keywords:** Affective computing, Anger, Contextualized rehabilitation technology, Emotion, Emotion recognition, Physiology, Photoplethysmography, Pervasive computing, Traumatic brain injury, Wearable

## Abstract

**Background:**

Anger dyscontrol is a common issue after traumatic brain injury (TBI). With the growth of wearable physiological sensors, there is new potential to facilitate the rehabilitation of such anger in the context of daily life. This potential, however, depends on how well physiological markers can distinguish changing emotional states and for such markers to generalize to real-world settings. Our study explores how wearable photoplethysmography (PPG), one of the most widely available physiological sensors, could be used detect anger within a heterogeneous population.

**Methods:**

This study collected the TRIEP (Toronto Rehabilitation Institute Emotion-Physiology) dataset, which comprised of 32 individuals (10 TBI), exposed to a variety of elicitation material (film, pictures, self-statements, personal recall), over two day sessions. This complex dataset allowed for exploration into how the emotion-PPG relationship varied over changes in individuals, endogenous/exogenous drivers of emotion, and day-to-day differences. A multi-stage analysis was conducted looking at: (1) times-series visual clustering, (2) discriminative time-interval features of anger, and (3) out-of-sample anger classification.

**Results:**

Characteristics of PPG are largely dominated by inter-subject (between individuals) differences first, then intra-subject (day-to-day) changes, before differentiation into emotion. Both TBI and non-TBI individuals showed evidence of linear separable features that could differentiate anger from non-anger classes within time-interval analysis. However, what is more challenging is that these separable features for anger have various degrees of stability across individuals and days.

**Conclusion:**

This work highlights how there are contextual, non-stationary challenges to the emotion-physiology relationship that must be accounted for before emotion regulation technology can perform in real-world scenarios. It also affirms the need for a larger breadth of emotional sampling when building classification models.

## Background

Anger dyscontrol (i.e., difficulty controlling episodes of anger) is a prevalent issue after brain injury that can have damaging consequences on self-care and relationships [1–3]. With the proliferation of wearable sensors and pervasive computing, there is new potential for technology to facilitate behavioural therapy supporting emotion regulation in the context of daily life [4]. *Affective computing* is the area of research that explores how technology interfaces with emotional phenomena [5]. Particularly, research into physiological affect recognition, or the ability for computing devices to detect emotions via bodily signals, could enable individuals with traumatic brain injury (TBI) to self-manage anger dyscontrol events before or as they occur. This potential, however, is predicated upon finding markers within physiology that are able to distinguish the changing emotional states of an individual, and for such markers to generalize to real-world settings. Of interest are markers from the autonomic nervous system (ANS), as most portable physiological sensors monitor aspects of the ANS (e.g., heart rate, respiration, electrodermal activity, etc.) [6, 7].

This research aims to further explore the potential of one ANS signal: photoplethysmography (PPG), and its ability to differentiate anger within a heterogeneous population. In this background, we begin by outlining the indicand-indicator framework to understand the emotion-ANS relationship, then we present a brief overview of TBI and its implications on the emotion-ANS relationship, later we explore PPG as a candidate physiological signal, and then review prior work on PPG related changes associated with anger. Lastly, we end this section with our motivation for studying a heterogeneous emotion-physiology dataset, particular in how it expands the spectrum of emotion regulation variability across individuals.

### Emotion and ANS physiology

**Fig. 1:**
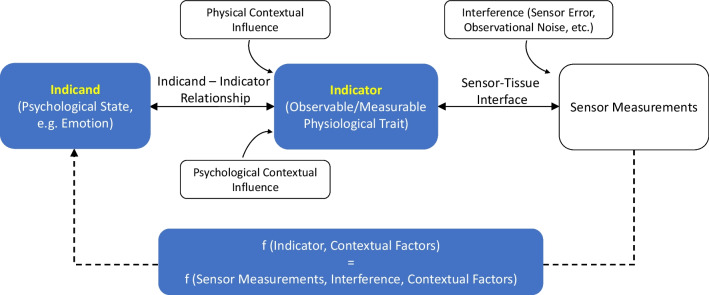
Emotion-Autonomic Nervous System (ANS) (indicand-indicator) relationship, showing potential influencing pathways and contextual noise. The indicand is inferred from a function of the indicator (inclusive of sensor measurement and interference) and contextual factors

The ANS regulates bodily functions of the internal organs and has been known to relate to emotional change [8, 9]. This psychophysiological relationship has been previously framed with three components: (1) the ***indicand***—an abstract psychological characteristic that is of interest but difficult to measure (i.e., emotion); (2) the ***indicator*** – a measurable physiological characteristic that is related to the indicand (e.g., heart rate); and (3) the ***indicand-indicator relationship***, which describes how changes in the indicand relate to changes to the measurable values of the indicator [10, 11]. Notably, the indicand-indicator relationship can range from a null relationship, to simple one-to-one linear mappings, to complex nonlinear mappings that have many known or unknown external influences on the indicator values [11–13]. Under this framing, ANS physiology is both influenced by the emotion proper, and external influences that relate to either the non-emotional context (e.g., body posture, temperature, physical activity, etc.) or emotional context (e.g., behavioural, and mental demands regulating emotion), see Fig. 1.

For affect recognition applications, the challenge then is two-fold: (1) to distinguish when an indicator physiological signal is being influenced by emotion, as opposed to other contextual bodily changes; and (2) to identify which and to what extent an emotional state is present in the current indicator value. This characterization aligns with the view that emotions are subjective, internal feelings that have qualities of intensity, temporal presence (e.g., onset, peak, decay), and differentiation (i.e., different types of feelings) [14–16]. Additionally, as opposed to underlying mood, emotions have event-relatedness and are driven by either exogenous (i.e., external sensory pathways) or endogenous (i.e., internal thought or memories) cues [11]. Such a stance is in agreement with clinical descriptions of anger dyscontrol, in that it is a spontaneous event, driven by either an individual’s external situation or their internal memories/thoughts [1].

### Traumatic brain injury

Traumatic brain injury (TBI) is caused by blunt or penetrating trauma to the head. This trauma can cause a wide array of focal and diffuse damage to the brain, including: cerebral contusions, diffuse axonal injury, and shearing strains [17, 18]. Additionally, secondary damage due to bleeding and intra-cranial pressure can result in further damage after the initial impact [19]. As there are a wide array of injury mechanisms, chronic TBI is considered a complex heterogeneous injury, where each patient has differing impairments [20]. Functionally, chronic TBI can have a persistent negative impact on the basic sensory/motor systems, cognitive functions (such as memory, language, attention, visuospatial, executive functions, etc.), mood/emotion regulation, and personality. The long-term effects of TBI may result in difficulty returning to school/work, loss of social relationships, reduced mental health, and difficulties completing tasks of daily living [21].

Despite TBI being a heterogeneous injury, in that no two injuries are alike, the prevalence of control issues related to anger remains high throughout the TBI population [1]. Anatomically, it is thought that damages to the hypothalamus, amygdala, septum, anterior temporal lobe, frontal cortex, and prefrontal cortex have a role in anger or aggression tendencies. Specifically, frontal lobe injuries are believed to be linked with loss of control of emotionally charged behaviour [22]. An added complication is that TBI injury may also influence ANS reactivity to emotion stimuli, with TBI found to generally have lower physiological reactivity to emotion than healthy controls [23]. However, this evidence must be interpreted with care, as in some cases the criteria for controls were only matched by demographic attributes (i.e., gender, age, education) and not physiological attributes that could bias ANS measures.

### Photoplethysmography

Although a number of ANS signals have been explored for affect recognition (see reviews [6, 7, 24]), photoplethysmography (PPG) remains of high interest due to its low-cost, prevalence, and compact form in wearable devices (e.g., smart watches, headbands, etc.). This portable form aids the potential for PPG to be used in *contextualized rehabilitation* applications: an approach that aims to integrate rehabilitation strategies into real-world context [4, 25]. Furthermore, research into the PPG waveform is still evolving, as there is clinical acknowledgement that its relationship with the ANS is not yet fully mapped – thus, there is potential for new insights to be derived from this waveform [26, 27]. Hence, PPG's trade-offs between physiological indexing, technology feasibility, and context plausibility remain an area for further investigation [28]. It is for these reasons that PPG was chosen as the focus of this research.

**Fig. 2:**
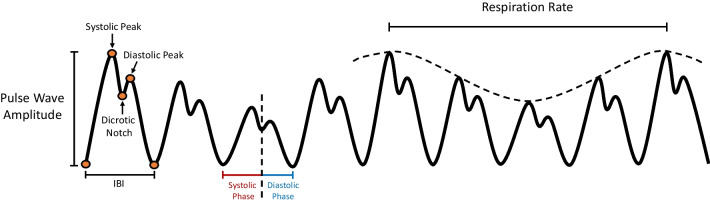
The photoplethysmography (PPG) waveform. IBI: interbeat interval

The PPG waveform (see Fig. 2) is created by either light transmission or reflectance through body tissue (i.e., skin, bone, blood, arterial/venous vessels), and is primarily influenced by the rhythmic fluctuation of blood through a tissue site [29]. PPG is a volumetric signal, that has a pulsatile component related to the blood volume of each heartbeat, and a quasi-static component that has been linked to underlying respiration, sympathetic nervous activity, and thermoregulation [26]. The PPG pulse has two phases: *anacrotic* – the rising edge of the pulse, related to systole; and *catacrotic* – the falling edge of the pulse, related to diastole. In healthy individuals with compliant arteries, a dicrotic notch is seen in the catacrotic phase and associated with a second elevated pulse. Since the recorded signal is a complex interaction of light with biological tissue, the PPG waveform varies depending on the sensor location (e.g., wrist, finger, etc.) and orientation of the tissue site with motion. Clinically, PPG has been used for physiological monitoring (e.g., blood oxygen saturation, heart rate, blood pressure, respiration), vascular assessment (e.g., arterial disease, venous assessment, tissue viability), and autonomic function measurement (e.g., heart rate variability, thermoregulation, blood pressure) [26, 27, 30]. Previously derived features for these clinical assessments include morphological, temporal, spectral, and nonlinear characteristics of the PPG waveform. Additionally, new time-series characteristics could be discovered that have further clinical value [27].

PPG sensing, however, is not without its challenges. Particularly, noise artifacts caused by motion (i.e., gross motor movement, tremors, coughing/yawning, etc.), skin displacement, skin blanching, and ambient light leakage at the sensor-skin interface, all impede the accuracy of physiological assessment [26, 31, 32]. Motion artifacts are especially challenging because the noise is typically in-band (i.e., overlapping the signal frequency), which complicates PPG noise separation techniques beyond simple frequency filters [31]. Furthermore, reproducibility of the PPG signal is affected by several factors including the sensor-tissue interface, ambient temperature, and daily variations in physiology either in or across individuals (i.e., posture, skin thickness/color, wakefulness, breathing, etc.) [33, 34]. Despite attempts to calibrate for these differences, standard normalization practices have not been established to account for such physiological variability [27]. Together, these challenges must be overcome before PPG can be fully realized in pervasive sensing applications, such as contextualized rehabilitation.

### PPG sensing of anger

The knowledge base of how PPG relates to anger stems predominantly from two fields of research: *psychophysiology* and *affective computing*. Both fields explore the indicand-indicator relationship with slightly different approaches to seek understanding. Psychophysiology forms an evidence base through repeated experimental trials, where the independent variable is the elicited emotion, and the dependent variable(s) are the observed physiological responses. Through repeated experimentation, psychophysiology researchers look for corroboration (usually correlative in nature) in the observed physiological responses to determine evidence of ANS response specificity for different emotions. PPG, as one such ANS measure, has been recorded over numerous psychophysiology studies, as well as its related influencing systems (i.e., cardiovascular, blood pressure, respiration). In the complementary case of affective computing, the indicand-indicator relationship is investigated through data modelling. Affective computing researchers compile large datasets of emotion-physiology pair examples, which are later interrogated by machine learning techniques to explore a mapping function from the observed physiological value (i.e., indicator) to an inferred emotional state (i.e., indicand) [7, 24]. The integrity of this mapping function is dependent on the sampling of emotion-physiology pair examples within the dataset. Therefore, larger, more thoroughly sampled datasets are needed to encompass and model the complexity of emotion-physiology in daily life [35]. Highlighted below are core findings related to PPG and anger.

### Psychophysiological response to anger

Psychophysiology studies of emotion typically comprise of a three-step methodology: (1) a baseline physiological measure of neutral emotion, (2) an induction to the target emotion with/without subjective confirmation, and (3) a response physiological measure for comparison to baseline. Within this framework there are a diversity of ANS physiological measures (e.g., heart rate—HR, heart rate variability—HRV, blood pressure—BP, etc.), emotion induction techniques (e.g., film, personal recall, real-life manipulations, picture viewing, etc.), and measured physiological response intervals (i.e., 60- to 30-s areis most common, but has range from 0.5- to 300-s intervals [8]). The diversity of these techniques highlights a key challenge in compiling an evidence base within this literature, as variations between response patterning may be attributed to experimental methodology differences, in addition to the elicited emotion and context [36].

In a review of 134 studies, Kreibig [8] found *directional stability* for ANS response patterning to anger as defined by the existence of directional change from a neutral baseline in a majority of studies. Relevant to PPG, cardiovascular responses showed reciprocal sympathetic activation and parasympathetic inhibition, as indicated by an increase in HR, decrease in HRV, variable systolic blood pressure (SBP), increased diastolic blood pressure (DBP), increase in total peripheral resistance (TPR) and decrease in finger pulse amplitude (FPA). Additionally, anger led to greater respiratory activity, as seen though increased respiration rate (RR), and decreases in inspiration time (T_i_) and expiratory time (T_e_). Of note, the emotion of fear had a similar ANS response patterning to anger, and only TRP was found to differentiate the two with an increase in anger, and a decrease in fear. An important caveat is that although these aggregate directional changes existed, there were occurrences when anger produced an ANS response that was counter to these aggregates. Kreibig suggests that these opposing variations may be due to sub-forms of anger that deviate from the common modal response pattern, as various types of induction techniques could produce different motivators for anger (e.g., approach-oriented anger, withdrawal-oriented anger, anger in defense of other, anger in self-defense, indignation, etc.) [8].

When looking beyond directional change and into the intensity of ANS response, meta-analysis reviews have produced similar results, supporting evidence of ANS change when emotion is present. Cacioppo et al.'s [37] review of 22 studies found that anger produced greater HR acceleration compared to disgust and happiness. Additionally, when comparing anger to fear, anger produced: higher DBP, lower HR, larger TRP, lower stroke volume (SV), and larger finger pulse volume (FPV). A concluding remark from this review was that discrete emotions (i.e., happy, sad, anger, fear, disgust) were not easily differentiated from ANS response alone – however, Cacioppo et al. [37] also noted that their analysis examined statistical differentiation via univariate physiological measures and not in terms of multivariate ANS response patterning. In a more recent review, Siegel et al. [38] compared 204 studies with multilevel meta-analysis and multivariate pattern classification analysis (MPCA). Anger resulted in an increase of mean effect size (change from neutral baseline) for HR, DBP, SBP, RR – however for all these measures, the results were largely heterogeneous (i.e., large variability in effect size between studies). With MPCA, Siegel et al. [38] found low classification performance (31.5% accuracy) in differentiating types of emotion using ANS measures. Furthermore, the authors proposed that the variability in effect sizes makes it difficult to claim that there exists *one unique* ANS modal response (i.e., fingerprint) for each emotion type, rather they argue that ANS responses are contextually modulated and should be viewed from a *populations perspective*, in that expected ‘archetypal’ ANS responses to a certain emotion are population aggregates, and that variability within the population is a definitive aspect of response patterning. In contrast to Kreibig's view of emotion sub-forms [8], a populations view claims that there are no true modal/ ‘archetypal’ ANS responses even within sub-forms of emotion, as each expression is context dependent [38].

This brief review of psychophysiology literature highlights several points that are relevant for understanding the indicand-indicator relationship of anger-PPG: (1) There is substantial evidence showing that anger induces *some form* of ANS response, particularly in the cardiovascular (i.e., sympathetic activation, parasympathetic inhibition, blood pressure changes) and respiratory systems, both of which are influential systems to the PPG waveform. (2) Although aggregate results may show *directional stability* in ANS response or an increase in mean effect size, there exist counterexamples (i.e., opposing directional response) and heterogeneity (i.e., non-standard ANS response patterning). While the cause of this ANS response variability is heavily debated within psychophysiology literature (e.g., spontaneous error, sub-groups of emotion [8], or populations hypothesis [38]), the existence of variability adds complexity to understanding the anger-PPG (indicand-indicator) mapping. (3) A large number of studies in psychophysiology literature tend to focus on laboratory environments, healthy individuals, and ANS change from neutral baseline. Such experimental constraints may limit the true complexity of the anger-PPG relationship as it exists in daily life (e.g., other environments, heterogeneous population differences, ANS response from various moods/emotion other than neutral emotion, transitions between emotions, etc.). This is particularly relevant for the TBI population, as there may be impairments that influence the psychophysiological relationship: such as deficits in emotional empathy that have been observed to alter physiological responsivity when compared to healthy controls [39, 40].

### Modelling PPG to emotion

Within affective computing literature, PPG has been used as an indicator signal for numerous studies (e.g., [7, 11, 24]), either on its own or in conjunction with other physiological signals in order to differentiate emotional states. Typically, this involves a process of segmenting (i.e., windowing) streams of physiological data, deriving features from these segments, training a learning model to differentiate how these features map to emotional states, and then testing the performance of the model on unseen/out-of-sample physiological data. Studies have ranged in their application of PPG features, from known physiological features within medical literature (e.g. [41],), to exploratory features (e.g. [42]), and automated feature generation (e.g., [43] defined under blood volume pulse—BVP) with positive differentiation results—supporting the idea that PPG has characteristics, including more that could be discovered that benefit the emotion classification task.

However, not all studies can be interpreted with optimism. Concerns have been raised about the transparency of the accuracy metric when applied to imbalanced emotion datasets [44], and questionable rigour of holdout test sets that may have artificially inflated some study results [7]. Reported classification accuracies have ranged between 40 and 94%, however this is highly dependent on the dataset at hand, and modeling approaches cannot easily be compared between datasets [7]. Additionally, as Kreibig [11] notes, many affective computing studies have focused on the problem of emotion differentiation (i.e., physiological signals that always co-occur with emotion), rather than emotion detection, where physiological signals exist in all emotion/non-emotion contexts. Emotion differentiation is a stepping stone to the larger real-world problem of emotion detection. To advance our understanding of the emotion detection problem, emotion-physiology datasets with greater contextual variation are needed.

A further challenge when interpreting affective computing literature centers around the issue of emotion sampling within a dataset and generalization from that sampling. Numerous studies and prominent datasets have constrained sampling methods, typically utilizing one form of emotion elicitation (e.g., video or standardized pictures) to represent an emotion class [7, 24], when it has been previously reported in psychophysiology literature that different elicitation methods for the same emotion could evoke different ANS responses [36]. Furthermore, few emotion specific datasets (i.e., non-mood, non-stress focused) have explored multi-day sampling for individuals due to the challenge of labelling these transient emotion events in everyday life (i.e., naturalistic studies tend to use coarser sampling methods such as experience sampling, that are not well suited for capturing the dynamics of transient events [7, 45]). The few studies that have explored this transient emotion space have noted the influence of daily confounds and the need to build adaptive systems that can address these dynamics [46–48]. Finally, the TBI population is underrepresented within affective computing datasets, thereby limiting our understanding of how population heterogeneity could influence indicand-indicator relationships.

### Research gap

In summary, there are several factors motivating this research. First, PPG remains a high interest candidate signal, as its relationship to multiple physiological systems (e.g., cardiovascular, respiratory) holds promise for its use as an indicator signal to an emotion indicand. Second, there is a need to further explore daily and contextual changes in emotion-physiology datasets, as these will be encountered in real-world applications. Although naturalistic datasets attempt the breadth this exploration, natural immersion comes at the expense of accurate temporal labelling of emotion. As such, laboratory studies with diverse elicitation methods (i.e., both endogenous and exogenous, with additional physical context) may provide a middle ground between immersive elicitation and temporally accurate tracking of emotion phenomena. Third, the TBI population remains understudied for emotion-physiology (indicand-indicator) relationships, as no affective computing datasets (to our knowledge) have included this population.

Therefore, in line with the motivation to develop a wearable device that aids the self-management of TBI anger dyscontrol—the goal of this study was to establish groundwork feasibility for PPG's use as an indicator signal to anger within a heterogeneous population. Specially, we sought to understand how PPG varies as an indicator signal when exposed to a variety of contextual changes (i.e., individual differences, emotional changes, and day-to-day variability in physiology), and to examine the stability of the anger-PPG (indicand-indicator) relationship for differentiating anger from non-anger across such contexts.

The choice to include both TBI and non-TBI individuals within this dataset was motivated by two factors: (1) the desire to capture a broad range of emotion regulation capacity, thereby sampling its possible effects on the indicand-indicator relationship; and (2) sampling for potential transfer learning between individuals. The second point is especially important for affective computing applications in clinical populations, as the labelling of subjective emotion data for these populations is time-intensive and may face large practical constraints. Hence, if there is useful information gained from a non-clinical population, this may expedite the development process.

### Research questions

1. *Indicator variability:* How does PPG vary across individuals (including demographics), day-to-day changes, emotions, and variability in emotion regulation capacity (as approximated by TBI status)?
2. *Indicand-indicator relationship:* What characteristics of PPG are able to differentiate anger from non-anger across individuals and day-to-day changes?
3. *Application design:* How can the above findings be integrated into the design of an anger classification model for an emotion regulation application?

## Methods

**Fig. 3:**
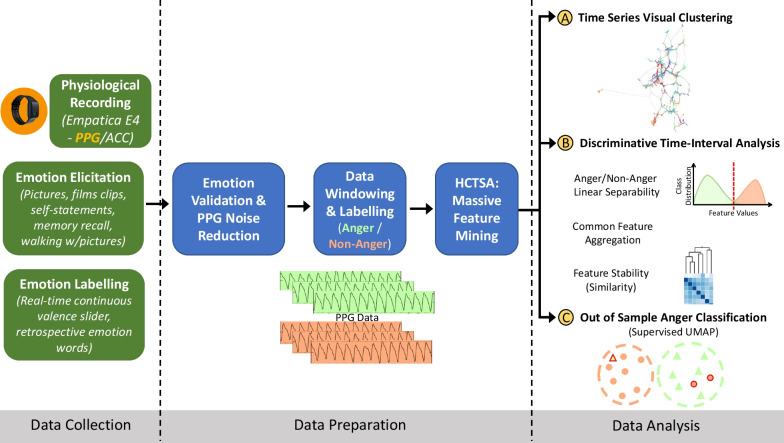
Overview of study methods. PPG: Photoplethysmography; ACC: Accelerometer; HCTSA: Highly comparative time series analysis; UMAP: Uniform Manifold Approximation and Projection

Figure 3 summarizes the methodological approach of this study. We begin this section by describing our emotion-physiology dataset, the data preparation steps, and finally our data analysis approach. The three research questions are explored respectively in our data analysis by: (A) time-series visual clustering, (B) discriminative time-interval analysis, and (C) out-of-sample anger classification.

### Dataset description

The following subsections describe the collection of the Toronto Rehabilitation Institute Emotion-Physiology (TRIEP) dataset. Using Picard et al.'s five factor guidance for high quality affective computing datasets [46], TRIEP was defined as having the following criteria: both subject- (i.e., endogenous) and event-elicited (i.e., exogenous) content, laboratory setting, internal feeling, open-recording, and known experimental emotion purpose. While the most naturalistic datasets are done in a real-world setting, with hidden-recording, and hidden experimental purpose, this dataset attempts to strike a balance between naturally elicited emotion and the temporal accuracy of emotion labels. Real-world datasets often struggle with defining the *exact* moment of emotional events, as it is difficult to continuously label subjective internal feelings beyond an experience sampling method [49]. Laboratory datasets, which have a variety of emotion induction methods, can still provide benefit for the affective computing field as they permit high-quality continuous labels, which allows for further interrogation into the temporally evolving emotion-physiology relationship. In line with the aims of this research, TRIEP was created to be a highly heterogeneous dataset in terms of participants (including clinical population), emotion induction material (i.e., endogenous/exogenous driven), and multi-day physiological confounds (i.e., day-to-day physiological changes and physical exercise).

### Dataset participants

Participants were recruited from two groups: (1) healthy individuals (non-TBI) and (2) individuals who had sustained a traumatic brain injury (TBI). Prior to recruitment, study approval was received from the Toronto Rehabilitation Institute-University Health Network Research Ethics Board (protocol #15-9925) and the University of Toronto Research Ethics Board (protocol #33567).

- **Healthy individuals** were eligible for the study if they were: (1) 19 years or older, (2) fluent in English and (3) had no history of brain injury. Recruitment for healthy individuals was conducted through online demographic ads and print flyers.
- **Individuals with TBI** were eligible for the study if they were: (1) 19 years or older, (2) fluent in English, (3) had a clinical diagnosis of traumatic brain injury based on medical record or patient self report; (4) TBI symptomatology sufficient to warrant in-patient or out-patient rehabilitation; and (5) were at least five months or more post injury. Recruitment for eligible individuals with TBI was conducted through outpatient community clinics and a prior study volunteer pool at Toronto Rehabilitation Institute. Upon being screened eligible to the study, a clinical neuropsychologist reviewed the capacity for informed consent before approaching individuals with TBI or their substitute decision makers for study recruitment. After entering the study, individuals with TBI performed a neuropsychological battery to characterize their cognitive functioning (see Table 1). All study participants with TBI had two cognitive impairments in mild or greater, or at least one neuropsychological test in mild or greater. One participant with TBI had a severe GCS score of 3.

**Table 1:** Neuropsychological characterization of cognitive function (TBI group)

Test name	Raw score, mean (SD)	Norm score, mean (SD)	Function measured
Trail Making Test A	56.89 (45.36)	T = 37.22 (17.65)	Simple visual attention
Trail Making Test B	90.22 (48.23)	T = 44.89 (15.52)	Mental flexibility, set shifting
Symbol Digit Modalities Test—Written	45.40 (22.50)	Z = -0.76 (2.03)	Speed of mental processing
Symbol Digit Modalities Test—Oral	56.60 (24.18)	Z = -0.49 (2.07)	Speed of mental processing
Stroop—Word Reading	84.00 (24.56)	T = 38.50 (11.30)	Selective attention
Stroop—Reading Color Naming	67.30 (18.76)	T = 41.55 (12.49)	Selective attention
Stroop—Color Word	43.90 (13.72)	T = 48.90 (13.72)	Selective attention
Stroop—Interference	6.63 (6.44)	T = 56.45 (6.43)	Selective attention
Spatial Span Forwards	7.90 (1.97)	ss = 9.30 (3.06)	Visual attention
Spatial Span Backwards	7.40 (1.58)	ss = 10.40 (2.27)	Visuospatial working memory
Digit Span Forwards	9.10 (2.42)	%ile = 35.80 (32.92)	Auditory verbal attention
Digit Span Backwards	7.10 (2.69)	%ile = 55.10 (35.06)	Auditory working memory
Grooved Pegboard Dominant Hand	88.11 (30.99)	T = 34.67 (14.87)	Manual motor speed and dexterity
Grooved Pegboard Non-Dominant Hand	85.63 (14.37)	T = 42.13 (13.42)	Manual motor speed and dexterity
PAI—Anxiety	19.00 (10.95)	T = 52.40 (10.35)	Anxiety
PAI—Depression	18.70 (15.04)	T = 54.70 (16.07)	Depression
PAI—Mania	27.10 (12.04)	T = 54.40 (13.16)	Mania
PAI—Alcohol Scale	4.00 (3.62)	T = 48.50 (6.60)	Alcohol Misuse
PAI—Drug Scale	7.50 (15.61)	T = 51.00 (13.21)	Drug Misuse
PAI—Aggression Treatment Consideration	8.70 (5.87)	T = 42.70 (6.90)	Aggression

*PAI: Personality Assessment Inventory; ss* = *scaled score; SD* = *standard deviation; T* = *t-score; Z* = *z-score; %ile* = *percentile*

For both groups, individuals were excluded from the study if they had any of the following criteria: (1) history of psychotic disorder, (2) diagnosed developmental disorder, and (3) other known neurological conditions or system disease of the central nervous system (e.g., dementia, Parkinson’s disease, multiple sclerosis, Huntington’s disease, lupus, etc.). All participants had the capacity to provide informed consent or had a legal substitute decision maker. None of the TBI participants were known to be taking antidepressant medications.

In total 40 individuals participated in the data collection sessions, with 32 included for further analysis due to recording errors in the data collection process. Four healthy individuals were excluded due to a high ratio of non-corroborative emotion labels (explained in Data Preparation, ‘Emotion Validation’ below), one healthy individual was excluded due to sensor recording error, and three healthy individuals were excluded due to no anger elicited in at least one day session. Table 2 summarizes demographic information for the remaining participants in both groups.

**Table 2:** Demographics of participants

	Healthy	TBI
N (# Female)	22 (13)	10 (4)
Age, Mean (SD)	32.1 (10.3)	43.5 (12.9)
Years of Education, Mean (SD)	15.0 (1.6)	15.4 (1.7)

### Elicitation protocol

All participants completed two emotion elicitation sessions, each approximately 120 min long, and conducted on different days (at least one week apart) to minimize repetition effects. Within each day session, participants were *seated* in front of a computer screen and in a quiet room to limit external influences. The exception to this was during the emotion elicitation with walking section, when participants were asked to walk on the spot and the computer desk was raised to a standing position. All session prompts and elicitation material were cued through the computer screen. The experimenter was seated out of view of the participants. Prior to beginning each recording session, all participants completed a five-minute resting baseline. Approximately halfway through the elicitation session, participants were able to have a short break from protocol, and the five-minute resting baseline was repeated upon their return.

As individuals have their own subjective biases to emotional material [50, 51], a variety of elicitation methods were selected to maximize the potential of eliciting target emotions and to diversify the exogenous/endogenous factors driving each emotion. In total, five different elicitation methods were used: (1) pictures, (2) pictures while walking, (3) self-statements, (4) videos, and (5) personal recall. Each method offered a different length of elicitation and depth of natural immersion [52]. All methods were chosen to allow participants to self-rate their emotions in real-time, while experiencing the material. Target emotions were chosen from the four quadrants of the Circumplex Model of Affect [53], which is a commonly used representation of emotions based on positive/negative affinity (valence) and degree of activation (arousal). In this manner, we align to the view that elicitation material has affective quality that may perturb an individual's subjective feelings towards a target emotion. The choice of the four arousal-valence quadrants, as opposed to including a wider breadth of emotion experiences that can be describe with arousal-valance-dominance dimensions, was a practical constraint on timing due to possible experimental fatigue from a long elicitation protocol.

Below is a description of the elicitation methods used during each day session:

- **Pictures (4 sections per day, randomized order):** Two picture sets were used in this study: The International Affective Picture System (IAPS) [54] and the Geneva Affective Picture Database (GAPED) [55]. Both collections have been used extensively in human emotion studies to evoke brief, reactionary emotions. Each picture has normative ratings for their assumed valence/arousal response, and can either be presented on its own or in succession with similar pictures to elongate the emotional response. Within each day session there were four different picture elicitation sections. Each section contained 20 successive pictures, presented for six seconds per picture to target a specific quadrant of the Circumplex Model of Affect (e.g., high arousal, low valence).
- **Pictures while walking (4 sections per day, randomized order):** Similar to above, four different picture elicitation sections were presented to the participant to target a specific quadrant of the Circumplex Model of Affect (20 pictures, six seconds per picture). However, for these sections, the participant was asked to walk lightly in place while rating their emotions. This introduced contextual motion noise in the physiological sensor data.
- **Self-statements (2 sections per day, randomized order):** When many self-statements (e.g., “I feel rather aggravated now.”) are read in succession and ruminated upon, this has been shown to evoke a sustained emotional response. For this study, Engebretson’s Anger Induction (E-AI) approach [56] and the Velten Mood Induction Procedure for Elation (VMIP-E) [57] were used to elicit anger and happiness respectively. Both procedures contain phrases that escalate from neutral to high emotional intensity (E-AI contains 50 statements, VMIP-E contains 53 statements). Participants were shown statements at a pace of approximately seven seconds per phrase and asked to experience each statement as though it was their own thoughts.
- **Video clips (9 videos per day, fixed order):** Film and news clips were selected to evoke continuous and transitional emotions [58]. Prior to the study, a number of videos were pre-screen by 70 adults (mean age: 32.7 ± 11.4, age range: 19 to 60, 32 female), and rated for their valence and arousal content. A selection of these film clips was curated based on their consistency to evoke specific arousal/valence ratings across a population. Each day elicitation session used nine different video clips, which were counter-balanced in time (approximately 15-min) and target emotions (see Table 3).
- **Personal recall (2 sections per day, randomized order):** recalling past autobiographical events is a technique aimed at evoking specific emotions associated with relevant life situations [59]. Prior to each day session, participants were asked to write out two specific events, one that made them happy and the other angry. Within each elicitation session, these events were recalled internally for two minutes to elicit an emotion. Participants were asked to imagine themselves in the situation and relive their emotions. This procedure for prompting autobiographical events was based on the solitary recollection approach [60].

**Table 3:**
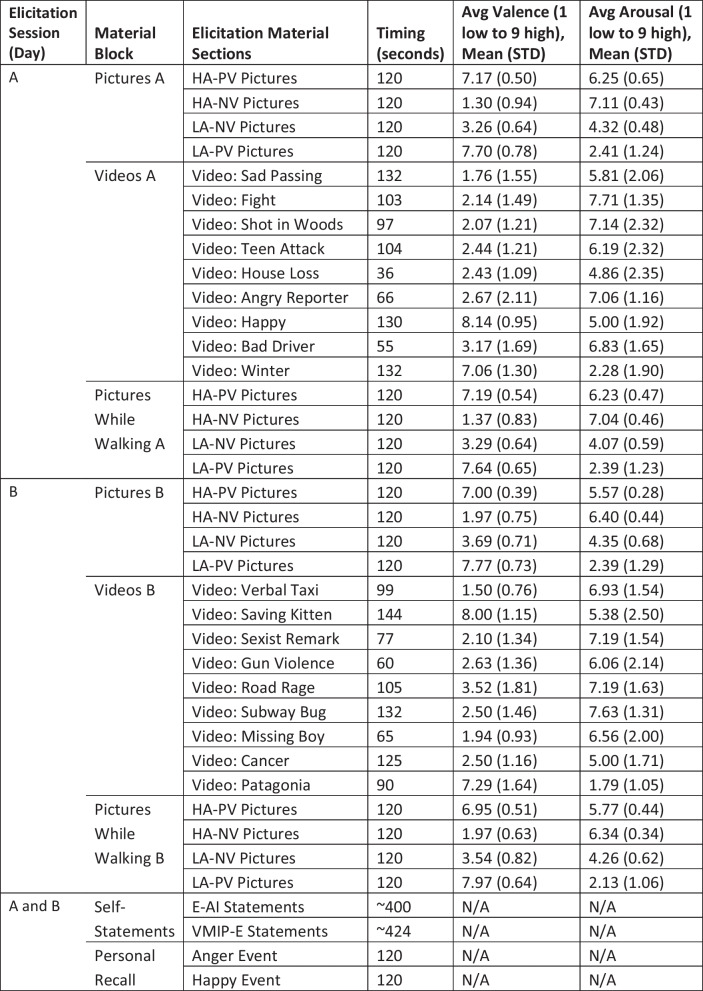
Summary of elicitation material timing and target normative scores

A summary timeline for each day session is presented in Table 3. With the exception of videos, all elicitation material were randomized within their given block. All emotion sections were preceded by a 30-s clip of neutral elicitation material (i.e., low stimulus figure) and followed by a 90-s clip of neutral elicitation material. The exception to this was the of the video section, where videos were played almost immediately after each other to add transitional complexity in the emotion data. The entire block of 9 videos was preceded/followed by the 30/90-s neutral stimuli.

### Outcome measure: physiological recording

For each day session, raw PPG signals and accelerometer data were recorded by the Empatica E4 sensor which was worn on the non-dominant wrist of each participant. The Empatica E4 is a commercial research device that captures PPG using a dual-red/green infrared source at 64 Hz [61]. Tri-axis accelerometer data was recorded at 32 Hz for a motion noise reduction algorithm.

### Outcome measure: emotion labelling

Labelling emotion is a subtle task, as requiring individuals to self-report their emotions may contaminate their current emotional experience [62]. To circumvent this, past researchers have relied on retrospective labelling (i.e., rating what was previously experienced moments before) or observer labelling (i.e., third-party observers rating an individual’s emotion based on facial or behavior changes). Both methods have the advantage of not conflicting with an individual’s current emotional state; however, they lack the ability to report the subjective emotional experience on a moment-by-moment basis. In retrospective labelling, individuals may not remember exactly what was felt throughout the time-course of an emotional experience, or they may have a recency bias towards the last felt emotion [62]. For observer labelling, there can be large discrepancies between raters who are evaluating the same individual, and not all emotional change may correspond to observable behaviors [63]. The choice of labelling technique is therefore a trade-off between multiple factors, where subjectivity and time-granularity may come at the expense of an increased rating task-load.

**Fig. 4:**
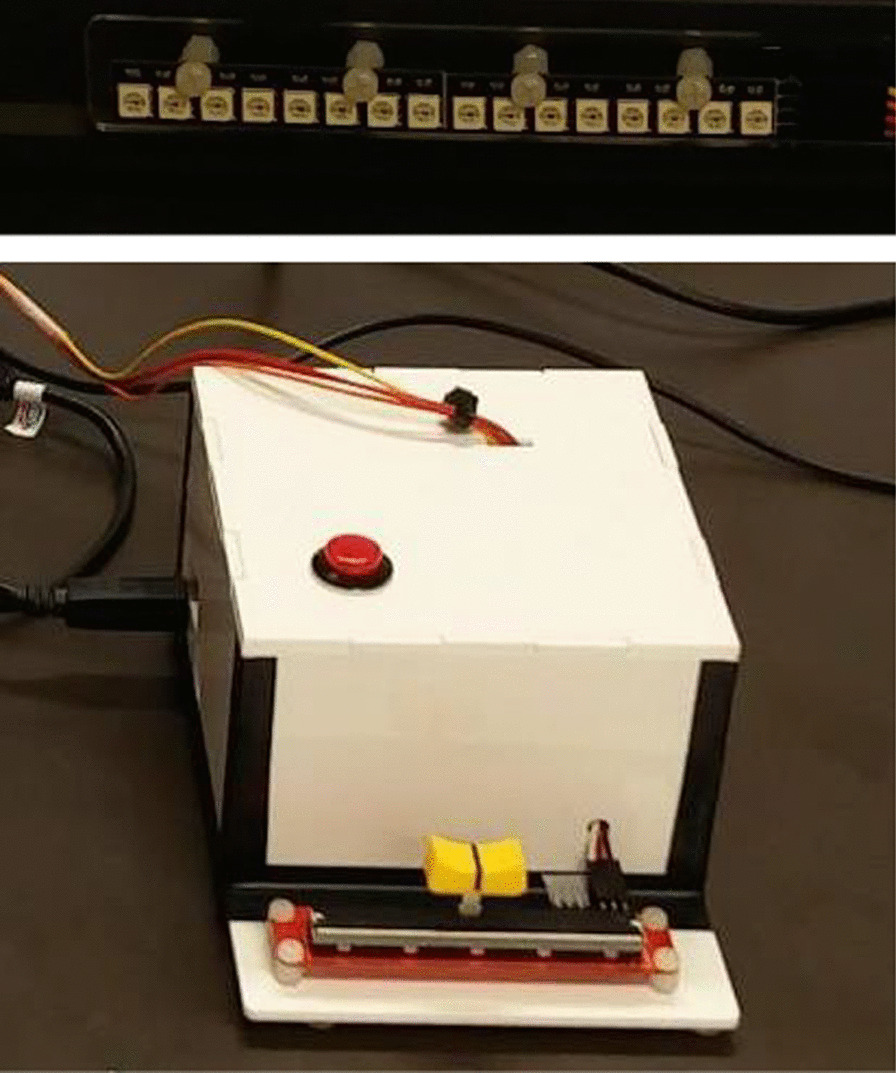
Custom valence rating slider. [Top] LED array [Bottom] slider interface

Nevertheless, past studies have found that subjective, real-time emotion changes can be tracked with the use of rating dials, sliders, and other continuous scales [64–66]. In comparison to discrete retrospective scales, such as the commonly used Self-Assessment Manikin [67], these continuous scales have been capable of recording changes in emotional intensity with high temporal accuracy. However, there is a balancing point with the use of such scales, as the more descriptive the rating (e.g., increasing the number of axes/dimensions rated), the more cognitively demanding the rating task becomes, which in turn distracts or fatigues from the current subjective experience [65, 68]. Moreover, patients with cognitive deficits may be more prone to error or mental fatigue as the complexity increases. Therefore, since this study involves individuals with TBI, a one-axis slider approach was chosen to lower dual-tasking demands. Additionally, to establish an *affective ground truth*, principles of emotional triangulation were applied, where multiple subjective labelling methods are used to corroborate each other [69]. In this study, participants rated their emotions with two complementary approaches:

1. **[Primary rating] real-time, continuous emotion slider:** A custom valence slider (see Fig. 4) was used with a small display that had 8 red LEDs on the left, and 8 green LEDs on the right. The position of the slider represented the intensity of valence in either the negative direction (left) or positive direction (right). As the slider moved position in either direction, the LEDs would light accordingly, as visual feedback for the user on their current subjective rating. When the slider was in the middle, the two LEDs in the middle would change color to a neutral yellow. This visual feedback was placed directly below the emotion elicitation material so that the individuals could simultaneously rate their emotion as they experienced the material. Participants used their dominant hand to control the slider scale.
2. **[Secondary rating] retrospective descriptive words:** Following each elicitation section (see Table 3), participants were asked to select one word from an arousal-valence figure [70] that best describes their overall emotion for that section. This was a method of confirming subjective internal emotion, as real-time valence response should corroborate with the quadrant of the chosen word.

Prior to beginning each day session, each participant received training, or a refresher on how to use the rating tools. The participant was informed that there was no right or wrong response to any of the materials, only that their rating should follow as closely as possible to their felt emotion. In each training, the participant received an explanation of the rating slider, a calibration of the slider extremes, and were given time to familiarize themselves with the retrospective rating words. Following this, the participant rated their emotions on two practice sections to become accustomed to the rating interface and to clarify any questions they had.

## Data preparation

### Emotion validation

For all participants, each emotion section was screened for corroboration between their retrospective verbal label and real-time valence ratings (e.g., an anger verbal response should correspond with real-time negative valence ratings). Elicitation sections that had disagreement between these values were removed from further analysis due to the uncertainty of the true subjective emotion. Following this, four participants that no longer had samples from all five elicitation strategies were removed from further analysis. Corroboration accuracy, as defined by the percentage of elicitation sections that had valid corroboration, over the total number of elicitation sections for each participant, was calculated to be: 91.23 ± 8.01% across the healthy group and 93.22 ± 5.55% across the TBI group.

### PPG noise reduction

Since minor movements can induce motion artifacts in PPG, a noise reduction algorithm was applied to the raw PPG signal to obtain a clean reference signal. To counteract motion effects, the raw PPG signal was filtered by a combination adaptive and notch filter as proposed by Zhang et al. [71], a current state-of-the-art for PPG heart rate (HR) estimation. Prior to filtering, all tri-axis accelerometer signals were re-sampled to 64 Hz to match the PPG sampling rate. A filter window of 10-s was chosen, with an overlap of 8-s, resulting in a 2-s recovered PPG waveform at every overlap. LMS-Newton parameters were set to *M* = 17, *μ* = 9E−5, *α* = 2E−4 and *δ* = 400. All filter code was implemented in Matlab R2018a [72].

Although the recovered PPG signal from Zhang et al.’s [71] algorithm can preserve key frequency and morphological characteristics of the PPG waveform, such as the dicrotic notch, it is possible that other important undiscovered characteristics may be lost in the filtering process. Indeed, noise filters have inherent information loss as they choose to pass through key characteristics and exclude others in the filtering process. A number of filters that prioritized frequency characteristics for HR extraction, often do so at the trade-off of losing morphological characteristics of PPG, such as amplitude changes related to respiration [73]. Due to this, the noised reduced PPG signal was used only as a reference signal in this noise reduction workflow. Raw PPG signals that achieved an adequate likeness to the clean reference signal were passed for further analysis. To calculate this, raw PPG signals that had a Pearson Correlation Coefficient above 0.6 to the reference signal (within the window frame), were passed.

### Data windowing and labelling

Various sizes of time-series windows have been used across psychophysiology and affective computing literature (typical ranges between 0.5- to 300- seconds [8]). Each window choice explores a different view of how emotion could relate to underlying physiology. For PPG, a shorter window investigates moment-by-moment local changes of near-term heartbeats, whereas a longer window is situated to explore more global cardiovascular (e.g., heart rate variability) and respiratory effects. To remain exploratory in nature, this study opted for medium-term windows with length of 70-s, and an overlap of 30-s. This medium-term approach is an intermediary balance between short- and long-term variations in the PPG waveform, with potential sensitivity to both. Each PPG window was labelled with its retrospective verbal label (translated into binary class of anger/non-anger), summary demographic labels (i.e., age, gender, participant ID, day session), and TBI status (i.e., a proxy for emotion regulation capacity).

For window labels, there was non-exclusive handling of different emotion elicitation strategies. That is, for example, the segmented windows from video elicitation material were labelled into the binary class or anger/non-anger, in the same way that windows from the other elicitation sections would be (i.e., self-statements, pictures, personal recall, etc.). This approach was taken because the final goal is a real-time system that could perform in real-life across a multitude of daily stimuli, elicitation complexity, and transitions. By aggregating these elicitation approaches together, our analysis attempts to mine for similar physiological patterning within them.

Each window comprised of a time-series vector of 4480 points (70 s * 64 Hz). On average, 87 ± 16 windows were extracted for each participant out of a maximum of 127 windows across the two days of sampling. The difference in windows extracted per participant was due screening from the PPG noise reduction step and identified non-corroborative sections from emotional labelling. In total, 2769 time series windows were extracted across all participants.

### HCTSA: time-series feature extraction

In line with the motivation that PPG may contain yet-to-be discovered characteristics that are applicable to emotion recognition, this study used an exploratory approach for feature extraction with the Highly Comparable Time Series Analysis (HCTSA) Toolbox (v0.98, implemented in Matlab R2018a [72]). HCTSA is a compilation of over 7500 time-series analysis techniques from the numerous fields of study that investigate time-series data. It includes techniques from statistical distribution, stationarity, correlation, frequency transforms, basis functions, model fitting, nonlinear dynamics, complexity/information theory, and other domain applications (including healthcare, e.g., HRV measures, etc., that have been used as “classic” affective computing physiological features) [74, 75]. The benefit of HCTSA is a systematic and unbiased feature discovery approach. By leveraging the wide-breadth of time-series domain knowledge, as opposed to manually curating time-series features, HCTSA can lead to the discovery of new features relevant to the classification task [76]. Additionally, in contrast to automated feature extraction techniques, such as neural networks or genetic programming, HCTSA features remain interpretable and non-dependent on network topology. Notable uses of HCTSA include emotional speech [75] and physiological signals (e.g., peripheral nerve firing [77], EEG non-linearity [78]).

HCTSA was applied to each time series window, resulting in a matrix of 2769 × 6697 (Time Series Windows × Features) after excluding time-series calculations that did not result in a real number (e.g., positive/negative infinity [79]). All features were then normalized across participants using max–min to 0–1. This linear transformation preserves the relationships between raw data values for further exploration.

## Data analysis

### Time-series visual clustering

To gauge how PPG characteristics vary with contextual changes, we constructed a visual landscape of this variation through time-series visual clustering:

After applying HCTSA, each of the 2769 time series windows is represented as a point in a high dimensional space, with the 6697 PPG features composing the dimensional axes of the space [77]. To interrogate relationships in a high dimensional space, visual analysis via dimensionality reduction is a useful tool for uncovering patterns and structure within raw time-series data [80]. Uniform Manifold Approximation and Projection (UMAP) is one such dimensionality reduction technique that aims to preserves local and global structure in the data, especially when compared to other dimensionality reduction techniques such as t-SNE [81, 82]. This preservation property is helpful when exploring relationships within the raw data that may otherwise be lost in other data transformations. UMAP performs dimensionality reduction by first constructing a high dimensional graph representation of the data and then optimizing this graph to a low dimensional space [83]. Within this optimization, key hyperparameters are selected that alter the weighting of global or local data preservation [84].

For this study, default UMAP parameters were used as *n_neighbours* = 15, *min_dist* = 0.1, and lower order dimension as 2. UMAP plots were constructed with visual edges intact, to discern whether neighbouring points were connected from the high dimensional space. Data points were labelled with participant ID, session day, TBI status, and demographic information (age/gender). For visual analysis, data points were examined for structure in clustering (e.g., group separation, ordinal gradation of labels), repeated patterns, outliers, and anomalies in the data [80]. Reported visual structure was tested for consistency on a variance of UMAP parameters (n_neighbours = 15, 30, 60, 100; min_dist = 0.1 to 0.3).

### Discriminative time-interval features

To find the features/characteristics of PPG that are best able to differentiate anger from non-anger, we first identify the top PPG features that separate anger from non-anger classes within each day session (i.e., time-interval)—then we compare the commonality and stability of these top features across all day sessions:

Using the HCTSA framework, each elicitation session was analyzed independently for linear discriminative features that separate anger from non-anger classes. As each daily session was treated as a distinct time-interval [76], comparisons could be made between different individuals and daily changes to gauge the stability of found discriminant features. For all features within every day session, a linear classifier was trained and tested on in-sample session data using the HCTSA framework [75], with classification accuracy as the output metric. The linear classifier determined how well a linear boundary separates anger/non-anger classes. Such an approach is akin to using directional change (i.e., increasing/decreasing physiological values from a baseline) for determining physiological reactivity, a common method employed in psychophysiology studies [8].

**Evidence of anger/non-anger linear feature separability:** Since many features (i.e., the 6697 valid features following HCTSA feature extraction) are tested for linear separability, there exist a possibility that certain features obtained a high classification accuracy by chance [75]. To account for this multiple hypothesis testing, a null distribution of classification accuracy scores was created for every feature in each session. This null distribution was made with the following steps: (1) randomly permutating the class labels assigned to feature values extracted from the time-series windows; (2) calculating the test statistic (i.e., classification accuracy scores from the trained classifier) for this permutation; and (3) repeating the process *k* = 1000 times to obtain a distribution of the null test statistic. Time-interval features were considered significantly linearly separable if they exceeded two conditions: (1) the 0.01 false discovery rate (FDR) test statistic from the null distribution; and (2) the majority vote baseline for *imbalance class accuracy* (anger/non-anger classes) calculated for each session. After all sessions were tested for their significant linearly separable features, individuals were categorized according to the existence of linearly separable features within their day sessions. Individuals fell into one of three categories based on their evidence of linear separability: (1) strong evidence (both sessions had at least 40 or more linearly separable features); (2) moderate evidence (only one session had at least 40 or more linearly separable features); (3) weak evidence (both sessions had less than 40 linearly separable features). A further population comparison between TBI and non-TBI groups was done with the Fisher Exact Test (Freeman-Halton extension) to compare the distributions into the linear separability categories between these groups.

**Common feature aggregation:** For every session, all significantly linearly separable features were sorted by classification accuracy. The top 40 of these features were then grouped into a combined pool across all sessions. A tally rank was made of the occurrences of each feature that shared tertiary similarity across individuals/daily sessions. Tertiary similar operations were defined by HCTSA naming, whereby similar time-series operations belong to the same algorithm family and have the same defining arguments [79]. For example, “*IN_AutoMutualInfoStats_diff_20_gaussian”* belongs to the overall correlation family of operations, the master family of statistics on auto-mutual information (AMI) from a time-series, and a tertiary operation family that explores AMI differences in a time-series using Gaussian estimation with a max delay of 20.

**Feature stability across sessions:** Prominent session features were then compared between all sessions to gauge feature stability. A cosine similarity score was calculated between pairwise sessions to determine the similarity of their top linearly separable features (up to 40 features, under tertiary HCTSA naming). Output of cosine similarity ranges from 0 (no similarity) to 1 (identical list). Each session was then plotted in a hierarchical clustering matrix, using average linkage based on Euclidean distance [85], to show similarity to other sessions in the data pool.

### Out-of-sample anger classification: UMAP metric learning

Finally, to explore how our findings can be integrated into the design of an emotion regulation application, we prototyped an anger classification approach:

This approach for anger classification was motivated by practical application constraints and findings from the prior empirical steps. The primary application constraints being: (1) non-stationary changes in a wearable setting, where physiological data is encountered newly each day—encompassing all changes from daily physiological variations and any contextual modulators of the indicand-indicator relationship; (2) difficulty in collecting training data for the indicand-indicator relationship—thus highlighting the need to leverage past labelled sessions from the same individual or related individuals (i.e., transfer learning). As such, this motivated an approach for a classifier that could be applied to newly unseen daily data, and which could leverage historical data from related individuals amidst any contextual variations that makes that data applicable/inapplicable for the new session at hand. As evidenced and explained later, our empirical findings highlighted the need for this context sensitive approach—specifically, it showed that there existed topologically separate subgroups within the data that had related PPG features for anger/non-anger separability.

The existence of these locally separate clusters in the PPG data space (i.e., data points that have PPG waveform characteristics that are largely distinct from each other), yet related in terms of PPG feature separability for anger, led to a hypothesis that there may exist a topology (i.e., manifold structure) which links together these separate clusters. In this sense, even though there is a broad heterogeneous landscape of PPG variation (whether by changes in individual, context, emotion stimuli, etc.), there could exist definable regions within this landscape which are attributable to the anger-class. The challenge then is using historical data to identify the topology of the anger class, and the subsequent transform that can group these anger clusters together for easier classification.

As UMAP is a strong approach for identifying topology in data (including sparse data), it can also be applied in a supervised way to learn a topology to separates classes. McInnes [86, 87], has describe this process for supervised UMAP learning as a metric learning approach, in that UMAP learns a transform that can map the original data space to a new metric that separates class labels. To accomplish this, UMAP intersects the simplicial sets of the unlabelled data topology and the labelled categorical metric. This intersection preserves topological connections in the original data only when they have the same class label, thus leading to an outcome of distinct topologies separated by class label. Thus, for categorizing new out-of-sample data, these new data points are embedded into the discovered metric based on their similarity to data points from the learned intersection.

Supervised UMAP was used with a leave-one-session-out cross validation (LOSOCV) approach. Training data were all day sessions excluding the test session in question (i.e., the training set encompassed day sessions from all participants, including the alternate day session of the individual under test). For this training data, a supervised UMAP was trained to map the original data space into a 10-dimensional new metric space. Essentially each axis in this new metric space acts as a learnt feature that optimizes class separation between anger/non-anger classes. A SVM (support vector machine) was then trained on this new metric space for classification. SVM parameters were chosen to be balanced weights with a radial basis function (RBF), optimizing for *C* (0.1–100) and *γ* (0.0001–10) via randomized grid search and fivefold CV. The trained UMAP metric space and SVM classifier was then used to classify the excluded test session data. Results were compared against a minority class baseline (as the rarer occurring anger class is of interest) for classification accuracy and F1 score. F1 score is an important outcome measure for imbalance class problems when there are low probability risk events. In our anger detection application, the minority risk event is important to identify, and hence should be weighted into the scoring metric. This is in contrast to classification accuracy (ratio of correct predictions to the total number of samples), where both classes are equally important in the classification score.

This division of training and test data was chosen to avoid temporal autocorrelation between the two datasets (i.e., there would be no adjacent windows between the training set and the test set, since these were from different days of data collection). This is a more challenging classification problem, but more akin to a real-world scenario of building classification models for an individual (which would likely be trained on previous day(s) of emotion-physiology data from a given individual, or other related individuals). Classification based on windows (as opposed to emotion event epochs) was chosen because this is a how a real-time system would view incoming data (i.e., there is a constant stream of new windows that would have to be classified for a real-time response).

## Results

### Time-series visual clustering

**Fig. 5:**
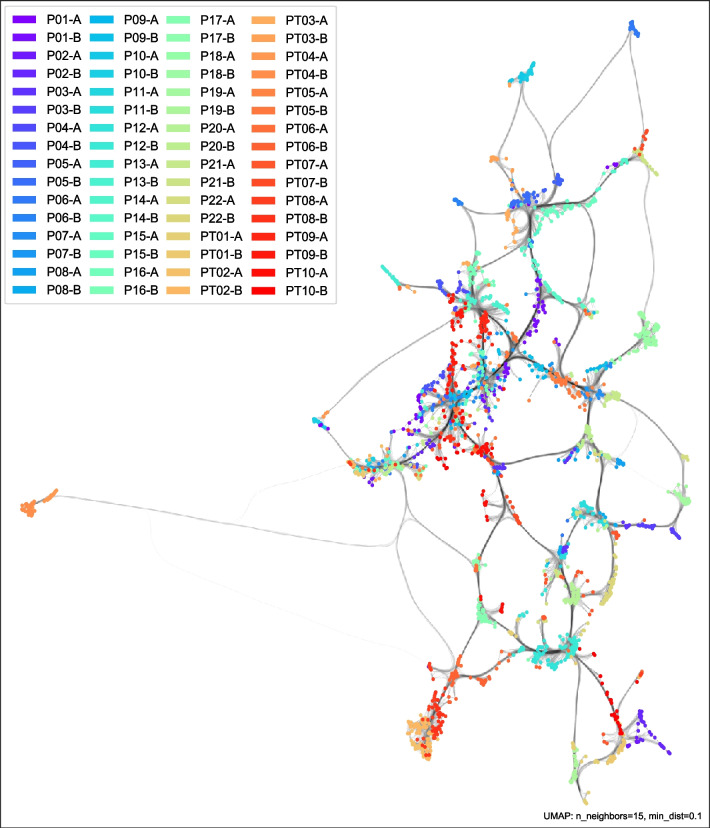
UMAP data visualization of entire PPG dataset. Colored by individual day session, where each participant had two day sessions ( e.g., P01-A is participant 01’s day A session). Dominant structure is due to individual differences. Edge connections show similarity between certain individuals and sessions. During visual cluster analysis individual points were checked for their actual labels to ensure color assignment were interpreted correctly

**Dominant local clusters:** From visual analysis (see Fig. 5), many PPG data points are clustered first within an individual's daily session, and second predominantly within close relation to the individual's other daily session cluster. Clusters tend to encompass a new region in the data space, showing that there are non-inclusive (i.e., non-stationary) changes to the PPG waveform characteristics between days. It is noted that for certain individuals, their between-session difference is greater than the difference to another individual's session, indicating a large contrast in daily physiology and/or sensor-skin interface. In total 19 individuals (59.5%) showed a near neighbour (i.e., edge connected) similarity between day sessions, 8 individuals (25.0%) within one session similarity, and a smaller proportion of 5 individuals (15.6%) having a larger deviation between daily sessions. Visual UMAP distances are not interpreted as an exact measure of difference, but a relative indicator of how closely data clusters are related.

**Fig. 6:**
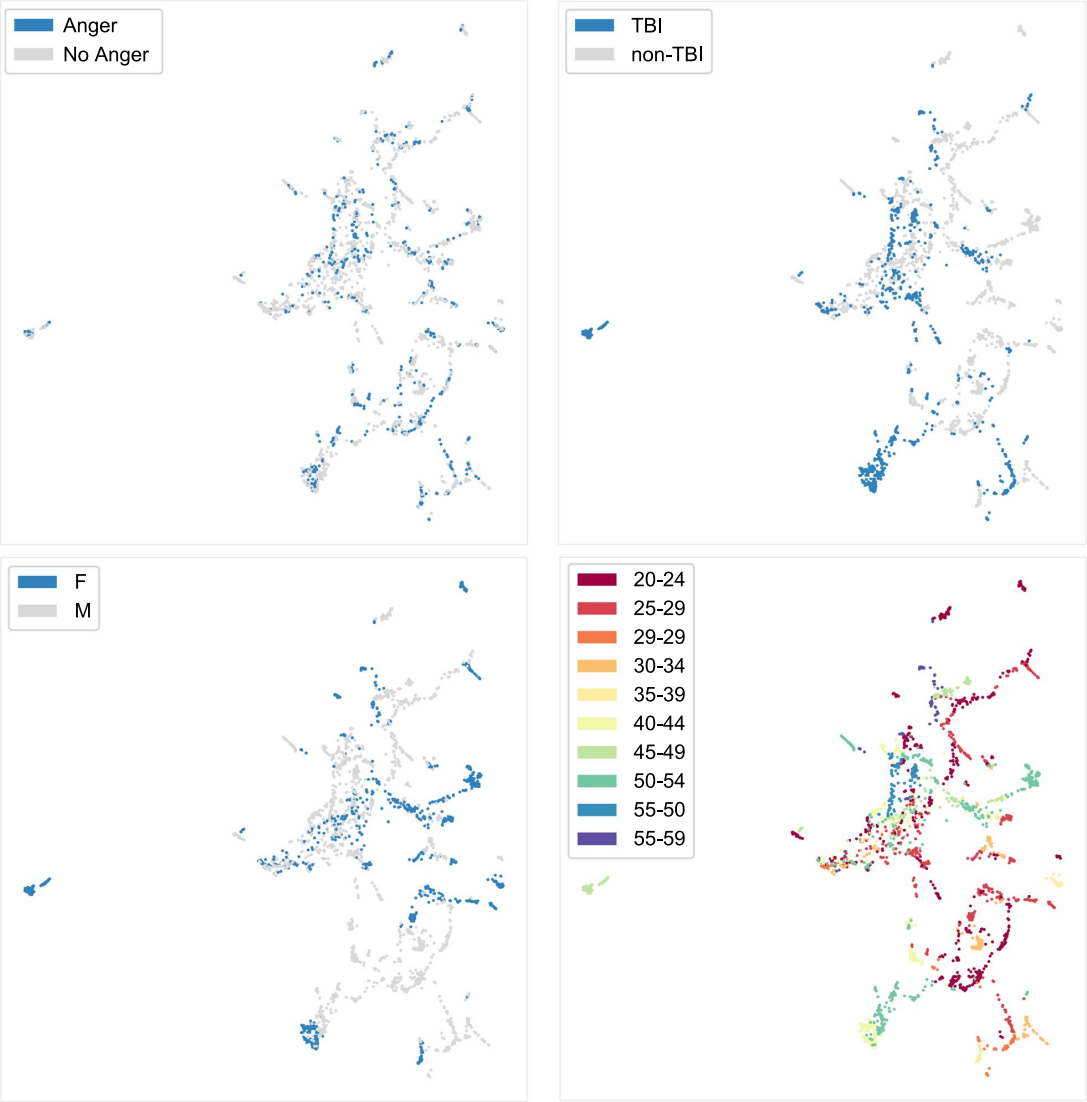
UMAP data visualization with stratification labels. Dense regions represent structure in data: [Top-Left] Anger, no anger; [Top-Right] TBI, non-TBI; [Bottom-Left] Gender; [Bottom-Right] Age

**Demographic influence on PPG (sub-groups):** It is noted that certain individuals appear more related than others by their cluster distances (taking into account edge connections). For example, PT02 and PT08 share a similar grouping. Both are in the TBI group, but of different gender and different age demographics. When exploring possible effects of demographics and TBI status on PPG (see Fig. 6), there does not appear to be a dominant clustering associated with demographic changes (i.e., gradation of age clusters as an ordinal category) or emotion regulation capacity (i.e., gradation of TBI status clusters from each other), instead we see sub-clusters related to subgroups of demographics or TBI status. Possibly, there exists other physiological attributes that were not recorded (e.g., body mass index (BMI), blood vessel sizing, skin tone, etc.), which could explain variations in and the existence of these sub-clusters.

**Influence of emotion on PPG:** In contrast to other individual or demographic related clusters, anger emotion clusters are smaller and more sporadic in the UMAP landscape (see Fig. 6). When exploring how emotion influences PPG, anger/non-anger classes appear to be highly mixed within the PPG data, indicative that among the aggregate of PPG features, emotions have a lesser influence on PPG than compared to individual differences. Despite this smaller influence, there exists minor local clusters of anger data points, which often relate to temporal auto-correlation of an emotion event.

### Discriminative time-interval analysis

**Evidence of anger/non-anger differentiation:** Of the 64 elicitation sessions conducted (32 individuals, 2 sessions each), 47 were able to identify at least 40 features which exceeded linear separability significance thresholds (i.e., 0.01 FDR and majority vote baseline). The remaining 17 sessions (26.6% of total) had partial features in this top 40 count that did not exceed the significance thresholds. These partial feature sessions accounted for 11 sessions of the non-TBI group (25.0% of group total) and 5 sessions of the TBI group (25.0% of group total).

Table 4 summarizes the occurrence of these sessions across participants, stratified by TBI status. Although linear separability is seen in most individual sessions, a proportion of individuals experience at least one session where psychophysiological differentiation between anger classes is more challenging, based on linear features alone. This proportion is similar between TBI status groups. Under the Fisher Exact Test (Freeman Halton extension) there is no statistically significant difference between the non-TBI and TBI distributions (*p* = 0.858).

**Table 4:** Strength of linear discriminating features (≥ 40) for anger/non-anger across participants

	Strong separability (both sessions)	Moderate separability (one Session)	Weak separability (no sessions)
*Non-TBI*	13 (59.1%)	7 (31.8%)	2 (9.1%)
*TBI*	5 (50.0%)	4 (40.0%)	1 (10.0%)
*Total*	18 (56.3%)	11 (34.4%)	3 (9.3%)

**Common top feature aggregation:** The most common family of operations that differentiate anger/non-anger across participant sessions are listed in Table 5. A number of these operations are based on modeling the structure of the time series and exploring the remaining error (i.e., residuals) that the model does not account for. In this regard, certain emotional states may show more stability throughout the PPG window than others, resulting in higher errors in the residuals when there is a variation from this state. Auto-mutual information, shape motifs, point density embedding, and power spectrum analysis are all sensitive to periodicity, as well as deviations from it when comparing two time series. In total, the number of shared features across all participant sessions is a small percentage – accounting for 17.2% of all sessions at maximum commonality.

**Table 5:** Common operation differentiating anger/non-anger across participants

Operation family	# Sessions	Description
MF GARCH_ar_P1_Q2	11	Fits a Generalized AutoRegressive Conditional Heteroskedasticity (GARCH) model to the time-series (order P = 1, Q = 2). Explores the appropriateness of model
IN_AutoMutualInfoStats_diff_20_gaussian	11	Automutual information statistics on the differences of the time-series. Uses gaussian estimation with a max delay of 20
MF_arfit_1_8_sbc	10	Fits Autoregressive (AR) models from order P = 1 to 8 on the time series. Optimal model is selected with Schwartz’s Bayesian Criterion (SBC). Statistics on model coefficients, final prediction error, and eigendecomposition, etc
SB_MotifThree_diffquant	8	Coarse grain motifs of an equiprobable three level alphabet (ABC) on the time-series differences. Outputs proportion of motifs ranging from word lengths 1 to 4
MF_ExpSmoothing_05_best	8	Fits an exponential smoothing model, by using half of the time-series as a training set to find the optimal smoothing parameter: alpha. Outputs fitting parameters and statistics on residuals
MF_AR_arcov_5	7	Fits an AR model of order 5 to the time series. Outputs parameters of model and residual analysis
MF_StateSpace_n4sid	7	Fits a state space model to the time series. Trains on first half of the time-series and predicts on second half. Outputs model parameters and statistics on residuals
SP_Summaries_fft	7	Power spectrum statistics using Fast Fourier transform (e.g., peaks, bandwidth, shape of cumulative sum, etc.)
CO_Embed2_Basic_tau	7	Properties of a point density embedding in 2D space (e.g., output of points near diagonals and geometric shapes)
WL_fBM	6	Wavelet estimation of fractional Brownian motion or Gaussian noise in the time series

**Fig. 7:**
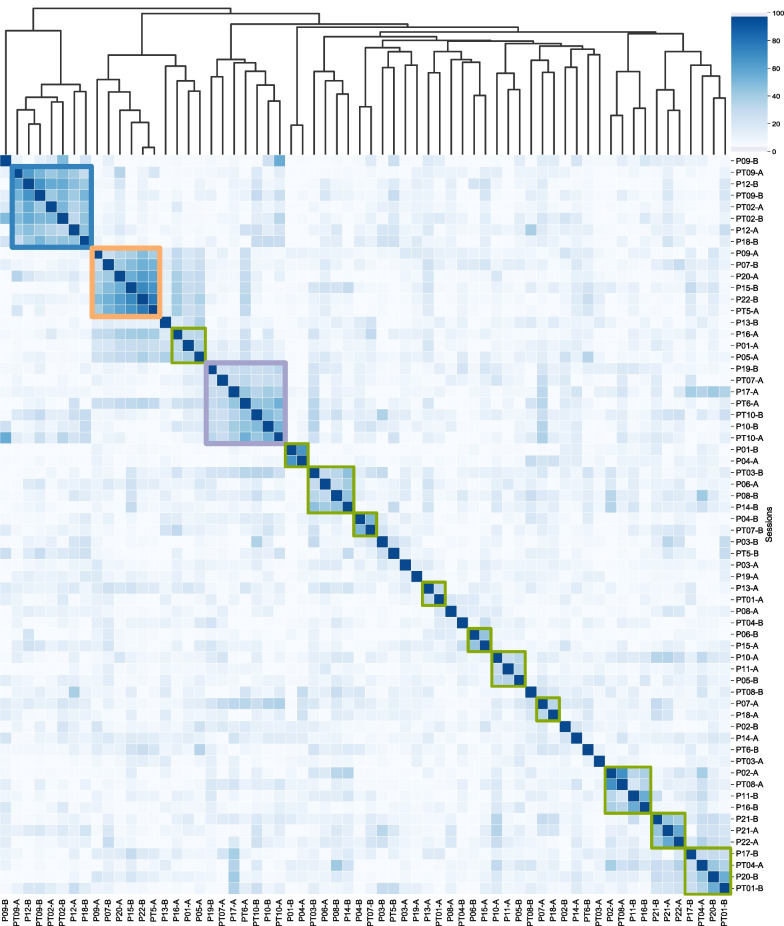
Comparison of top discriminating PPG features for anger/non-anger across sessions. Related feature sets are paired on hierarchical tree map. Dominant groupings are color marked with square boxes

**Feature stability across sessions:** Fig. 7 depicts a hierarchical cluster heatmap of all day sessions and the respective similarity between their top linear features that differentiate anger/non-anger classes. Sessions are highlighted in group boxes when their cosine similarity exceeds 0.25. It is noted that there are three dominant clusters, each grouping 6 or more day sessions. Interestingly, these cluster encompass a mix of TBI and non-TBI individuals, as well as sessions not from the same individual. Five individuals (i.e., P12, P21, PT02, PT09, PT10) had both of their sessions within the same cluster, indicative of similar anger-separable features that differentiated emotion across days. However, the large majority of individuals had different anger-separable features when comparing their two day sessions, as cosine similarities were less than 25%, indicating non-stationary changes in the emotion-physiology relationship (at least from a linear perspective).

**Fig. 8:**
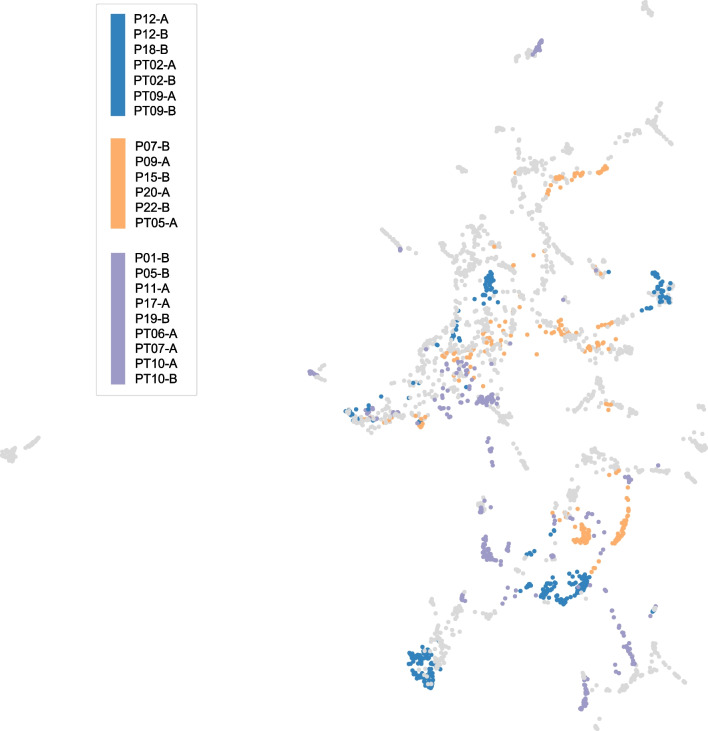
Dominant groupings of sessions with similar anger discriminating features on UMAP PPG plot

When mapped to the UMAP PPG space, these three dominant clusters form different sparse sub-groupings, indicating the challenge of gauging which features are important for emotion separability from PPG characteristics alone (see Fig. 8). There appears to be local regions in the dataset that prioritize different features for class separability.

### Out-of-sample anger classification

Anger prediction results from LOSOCV are reported in Table 6, with each participant reporting their averaged scores. Although an improvement can be seen in the UMAP + SVM accuracy scores above the minority vote baseline, F1 scores showed only moderate improvement for certain participants.

**Table 6:** Anger prediction scores (leave one session out)

	Baseline (Minority)		UMAP + SVM	
	*F1*	*ACC* (%)	*F1*	*ACC* (%)
P01	0.189	10.5	**0.255**	**52.3**
P02	**0.308**	18.2	0.222	**51.8**
P03	0.261	15.0	**0.359**	**68.8**
P04	**0.313**	18.6	0.286	**78.6**
P05	0.365	22.4	0.390	**70.6**
P06	**0.349**	21.1	**0.157**	**39.9**
P07	**0.543**	37.3	0.400	**54.2**
P08	0.545	37.5	**0.609**	**67.9**
P09	**0.417**	26.3	0.346	**64.2**
P10	0.393	24.5	**0.489**	**48.9**
P11	0.405	25.4	**0.516**	**55.2**
P12	**0.472**	30.9	0.412	**51.2**
P13	0.200	11.1	**0.264**	**51.9**
P14	**0.148**	8.0	0.100	**28.0**
P15	**0.382**	23.6	0.286	**65.3**
P16	0.286	16.7	**0.435**	**83.3**
P17	**0.606**	43.4	0.441	**50.0**
P18	**0.310**	18.3	0.291	**33.0**
P19	**0.613**	44.2	0.517	**50.0**
P20	**0.372**	22.9	0.206	**28.0**
P21	**0.544**	37.4	0.509	**68.1**
P22	0.355	21.6	**0.395**	**40.9**
PT01	0.316	18.8	**0.346**	**29.2**
PT02	**0.461**	29.9	0.396	**49.6**
PT03	0.242	13.8	**0.353**	**86.3**
PT04	0.460	29.9	**0.471**	**37.9**
PT05	**0.336**	20.2	0.074	**71.9**
PT06	0.482	31.8	**0.523**	**50.6**
PT07	0.191	10.6	**0.255**	**51.8**
PT08	**0.400**	25.0	0.372	**69.3**
PT09	0.466	30.4	**0.500**	**64.7**
PT10	0.437	28.0	**0.439**	**75.3**
Mean	**0.380**	24.2	0.363	**56.8**
SD	0.121	9.4	0.129	16.5

Bold values indicate higher score in comparison

## Discussion

In this discussion, we interpret the results as they relate to our research questions. Specifically, we explore: (1) how the PPG *indicator* signal varies through individual, day-to-day, and emotional regulation capacity (as approximated TBI status) changes; (2) how characteristics of PPG are able to differentiate anger/non-anger across these changes (*indicand-indicator relationship*); (3) and how the above findings can be integrated into the design of an emotion regulation system.

### Indicator variability: landscape of PPG variation

Time-series visual clustering identified core attributes associated with PPG variation across individuals and emotion. The most common cluster structure was attributed to daily differences between individual day sessions. Daily clusters of sensor data have been previously reported by Picard et al. [46] in the Eight-Emotion Dataset and inferred to be associated with daily physiological change, contextual emotional change, and sensor-skin interface variation. Similar to their dataset, it is uncertain from this experimentation the exact contribution of how these factors influence between-session differences (leaving a question for further exploration). However, unique to this study, it was noted that observed sensor differences between day sessions varied across individuals (i.e., differences in connected edge similarities in UMAP plotting), giving further evidence that the factors influencing daily sensor data have non-stationary properties.

A further structure was seen with daily clusters having more global groupings that encompass multiple sessions from an individual. In aggregate, it was common to see inter-individual separation of associated day clusters, which highlights how individual distinctiveness is a dominant attribute within the features of the PPG waveform. This is in agreement with biometric literature on physiological signals, where ECG (electrocardiogram), a related cardiovascular waveform, has unique attributes for personal identification [88]. Hence PPG is varying based on individual inter-subject differences, as well as, daily intra-subject changes.

Shifting broader from individual differences, there were occurrences of related session clusters between certain individuals (e.g., PT02 and PT08, among others). A plausible explanation for this may be the close physiological similarity between a smaller grouping of individuals, as these sub-groupings were not easily attributable to *only* demographic or TBI status (i.e., there exists other factors not recorded in this study, which cause the sub-grouping of similar PPG). Finally, when related to emotion, PPG showed smaller local clusters of related emotion types within a session, highlighting how variation in emotion is more of a local perturbation on top of the larger day/session and inter-individual differences.

### Indicand-indicator relationship: changing anger-PPG separability

Although all sessions were able to identify at least one PPG feature that linearly separated anger from non-anger classes, the number and types of these features varied from session to session. In total 17 sessions (26.6% of total) had weaker separability in PPG features according to the top 40 threshold. This variation could be related to the sampling of emotion classes as different endogenous/exogenous elicitation strategies may have evoked nuanced ‘sub-types’ of emotion (which has been previously inferred to cause variation in ANS response [8]), and/or due to uncontrolled daily physiological and psychological context influencing the ANS response (i.e., in line with the populations hypothesis for ANS response, where the ‘prototypical’ emotion-class response is an aggregate of contextual variations [38]). Globally, very few anger-separable PPG features were shared across all participants and sessions (see Table 5)—the most common of these PPG features encompassed a small subset of the study sessions (~ 17%), and included features related to: Generalized AutoRegressive Conditional Heteroskedasticity (GARCH) residuals, Automutual information based on Gaussian estimation, and Autoregressive (AR) models selected with Schwartz's Bayesian Criterion (SBC). It was noted that smaller sub-groupings of sessions, held more anger-separable features in common (see Fig. 7), showing a possibility of shared learning between these sessions.

The large variation in features that separated anger/non-anger classes between sessions calls to question whether prior normalization methods in affective computing are sufficient to encompass possible changes in the indicand-indicator relationship between sessions. Prior approaches of normalizing to a ‘neutral emotion’ physiological baseline [46, 89] tend to be based on the idea that a linear transform of indicator features can shift daily sensor values to be comparable to each other, and assumes features of interest still exhibit the same directional change within a daily session. These methods may have had modeling success due to conditions of the dataset, rather than its ability to generalize to wider emotion-physiology circumstances. For example, the Eight-Emotion Dataset made use of the Clynes protocol to elicit emotion over one individual, which is a protocol that has specific sequencing and imagery type for evoking emotions [46]. Hence, the sequencing structure and elicitation similarity may have preserved directional changes seen in certain ANS features across different days of data. As more variability is included in the elicitation protocol (i.e., changes to elicitation timing, sequencing, and medium of material—as akin to the various ways emotion could be triggered in real-life), these ANS indicator features could lose some of their directional structure, which was evident in this study through the differences in separable features between daily sessions, as well as reported ANS response variance in psychophysiology literature [38].

Interestingly, not all participants exhibited large variability in separable features between their daily sessions (e.g., P12, PT02, PT09, PT11). For these individuals, there were a larger set of common separable features that were shared across their daily sessions. This raises a key question for modelling: how does one determine when an indicand-indicator relationship is changing between day sessions, versus when it remains largely the same (i.e., separable features are held in common)? Such a question essentially seeks the *context* of the anger-PPG (indicand-indicator) relationship. Context, in this sense, is interpreted to mean the state-base property of the emotion-physiology (indicand-indicator) system, which is influenced by emotional/non-emotional changes [11].

In this study, it was observed that contextual similarity of anger-separable PPG features was difficult to determine from the characteristics of PPG alone (i.e., see Fig. 8, where related sessions are not grouped together based on the global UMAP mapping of PPG features and the transformed mapping learned with supervised UMAP performed poorly in out-of-sample classification). Hence, one would need to determine alternatives for deriving context from observed sensor (indicator) readings. A typical approach would be to include additional physiological/sensor modalities in the recording stream, hopefully serving as state variables/features that are sensitive to contextual states. However, the inclusion of more sensors does not guarantee that contextual states are observable from sensor data, and affective computing models may have to contend with the challenge of inferring unobservable states.

### Application design: challenges for anger detection

In an attempt to overcome daily non-stationary variations in the PPG sensor data, contextual local pockets of the anger class, and to learn from prior out-of-sample sessions—we proposed using supervised UMAP as a means to normalize/transform the PPG data space into one that was amenable for the anger detection task. Essentially, supervised UMAP would learn how to transform local regions within the original PPG data space into class defined regions in the new metric space (i.e., local regions in the original PPG data space would be attributable to a specific anger/non-anger class). There were several assumptions embedded into this approach: (1) a PPG data point had a fixed attribution to an anger class (i.e., the specific value of PPG features on each dimensional axis could only define one class attribution), and (2) unexplored regions in the PPG data space (i.e., non-stationary data variation that have not been observed by sensor readings), could be topologically attributed to a neighbouring class, and that the collected dataset was sufficient to define this attribution in unexplored regions.

Although UMAP + SVM showed improvements in accuracy over a minority vote baseline (see Table 6), the 56.8% mean accuracy is only marginally better than chance. F1 score did not show an improvement for UMAP + SVM over a minority vote baseline. This poor performance of the supervised UMAP approach highlights how challenging truly out-of-sample testing data is for emotion-physiology modelling. As seen from the PPG data structure (see Fig. 5), new daily (i.e., out-of-sample) sessions usually encompass distinct regions in the PPG landscape, meaning that their feature values are non-stationary/distinct from prior values in the dataset. A point of failure is when supervised UMAP has to approximate class regions in an unseen data space with few known data structures (i.e., other data points that define class regions). McInnes [87] has stated that supervised UMAP can fail when data structure is not sufficient, as seen in our attempt. However, the variation in F1 and accuracy scores across participants may point to better structural sufficiency for some of these participants over others (i.e., that there was enough overlapping data from prior sessions that could define class attribution in the test session). This leads to a further question of how much data across individuals and day sessions would be *sufficient* to define the emotion-PPG relationship within a given population.

The other point of failure relates to the assumption of fixed class attribution for specific PPG values. Similar to other non-stationary data streams, emotion-PPG data could experience real or virtual drift, which affects the underlying values of PPG data and their relationship to emotion class labels [90]. Virtual drift, where the marginal distribution *P(x)* changes over time has been previously reported in affective computing datasets and thought to be combated with dataset normalization [46, 89]. However, real drift where the posterior probability *P(y|x)* of emotion labels changes over time relative to PPG values, points to the contextual challenge of PPG alone defining anger classes (i.e., a specific PPG data point may be attributed to anger at one point in time and non-anger in another). In this case, non-stationary models would have to be developed to account for these changes, such as ensemble approaches that adapt to shifting days of data [47].

## Application design: implications for emotion regulation systems

Integrating the above, we highlight several implications for developing a future emotion regulation system:

1. **Non-stationarity of sensor data:** Similar to prior affective computing datasets that included multi-days of sampling (e.g. [46, 47],), we report that sensor data values exhibit day-to-day differences that must be accounted for across the population. A core challenge with this non-stationarity data is accounting for the effects of sensor-skin interface variation (influencing indicator values), and physiological/psychological contextual change (affecting the indicand-indicator relationship). Both effects may manifest in variations of the marginal and posterior probability distributions related to sensor values and labels respectively. Traditional static classification models are not sufficient to combat this challenge, and further exploration into adaptive systems or dynamic models are needed for real-world performance.
2. **Reliability of PPG as an indicator to anger:** Although for a majority of sessions (73.4%) there existed > 40 PPG features that could separate anger from non-anger, the inconsistency of these features and the existence of sessions that had limited separability is concerning. On the basis of PPG features alone, it was not seen that PPG could *reliably* act as an indicator signal to anger—in that more information is needed to ascertain *which and how specific* PPG features are able to separate anger/non-anger in a given situation (inclusive of the individual, emotional/non-emotional changes). We consider this information to be *state-variables* of the emotion-physiology (indicand-indicator) system, and there is a need to identify these contextual variables to improve PPG’s reliability as an indicator signal to anger.
3. **Defining emotion-physiology state context:** The existence of some individuals having similar anger-separable features between their sessions, and others that did not, supports the idea that there are contextual state variables that can differentiate these scenarios. Traditionally, the search for state variables takes the form of additional physiological or activity-based sensors/features (i.e., additional recorded data streams). However, when additional sensors cannot be added to a device or are insufficient to identify system states, other approaches for identifying hidden/unobservable system states could be applied (e.g., Takens' embedding theorem for nonlinear dynamical systems to recover state variables from the time-lag embeddings of a single variable [91]).
4. **Prospects for shared learning to individuals in the TBI population:** When considering the implication of emotion regulation capacity (as approximated by TBI status) on the indicand-indicator relationship, there appears to be some possibility of transfer learning between specific day sessions of individuals despite this difference. Notably, when comparing anger-separable PPG feature similarity between day sessions, there were several clusters that had both TBI and non-TBI day sessions (see Fig. 7), supporting the idea of a similar anger detection task within these clusters. As a specific example, if creating an anger identification model for an individual with TBI, e.g., PT09, and our out-of-sample test session is day A (i.e., PT09-A), then our interpretation is that pair emotion-PPG examples from day sessions P12-A, P12-B, PT02-A, PT02-B, P18-B, and PT09-B have high relevance for this anger identification training task (i.e., this includes learning from individuals without TBI).
5. **Data sufficiency:** Since physiological data are difficult and time intensive to label for emotions, real-world systems should endeavor to utilize forms of related data. Transfer learning between individuals and within an individual, is an important topic for affective computing. On a wider scale, as more data points are collected across individuals, emotions, and days, this evokes the question of how much data are sufficient before we see repeated patterns in the emotion-physiology system?

To summarize, our empirical exploration expands beyond describing “what” needs to be done, to proposing a possible “how” better modelling could be accomplished. In addition to obtaining and exploring a challenging heterogeneous dataset for affective computing (i.e., multi-day, multi-elicitation, and the inclusion of a clinical population), a major unique finding of this study was highlighting nuanced changes in emotional-physiology state context. Particularly, in Fig. 7, we showed how certain individuals had more stable discriminative features for anger than other individuals across their two-day sessions (i.e., whether their day sessions were related in cosine similarity for their top 40 discriminatory features). One common interpretation of this, is that physiological features that do not have stability across days of sampling would be poorly matched to an emotion inference task. However, underlying this interpretation is an assumption that emotion-physiology relationships need to exist in a co-occurring context. Meaning, if a trait of physiology is seen to change with an emotional phenomenon in one scenario (i.e., feature discrimination), and then seen not to change with the same emotional phenomenon in another scenario (i.e., no feature discrimination), that this is evidence of a weak or null emotion-physiology relationship. Stated another way, because of feature instability across day sessions, we would typically disregard features that do not show consistent discriminatory potential.

Yet, emerging work on dynamical systems, of which many naturally occurring systems are, offers an alternative explanation for this dichotomy: that both co-occurring (e.g., a correlative and discriminatory) and non-co-occurring (e.g., an uncorrelated and non-discriminatory) phenomena can exist within the same emotion-physiology relationship, as this relationship may alter through different states in time. Meaning that features that would typically be disregarded, could actually be valuable and just be changing in its relationship to emotion in time (i.e., there are periods of time that this feature is discriminatory to a certain emotion phenomenon, and periods of time where this feature is not discriminatory). This helps to offer a hypothesis of why some individuals are related in discriminatory features (Fig. 7) more than other individuals. I.e., this may not be a chance occurrence, but rather related to a temporal sampling of an emotion-physiology system that changes its internal relationships through time. One way to operationalize such a system, is by viewing it as a nonlinear dynamical system to define emotion-physiology state context.

Our empirical exploration also highlights how time-series approaches, could be used to mine further insights into the complexity of emotion-physiology relationships. We acknowledge that these methods are not without their limitations, yet with the heterogeneity and complexity of emotion-physiology data (especially when considering greater diversity in the sampling population, broader time-course sampling, and further contextual confounds when moving to real-world applications), the necessity of interrogative data-mining methods that help to explore this heterogeneity becomes ever more important. The methods presented in this paper focused on large time-series feature exploration, time-series visual clustering, and data mining. We view these as tools with different advantages and disadvantages for their use case, and invite affective computing researchers to consider how these and other time-series methods can inform our understanding of heterogeneous emotion-physiology datasets.

## Limitations

Although this study explores a highly heterogeneous emotion-physiology dataset, it nonetheless is limited by the sampling of that dataset. This study samples only a small subset of the overall population (22 non-TBI, 10 TBI), is constrained to two days of data across each participant, and selected for participants that had a propensity to anger within our elicitation protocol. Additionally, the choice was made to sample emotions from the circumflex model of affect, which limits the diversity of emotions (i.e., excludes emotions varying due dominance). As well, balancing of target emotion samples from the circumflex model was not controlled for, and may be an alternate reasoning to explain the difference of separable features between each day session. Further studies can expand upon sampling the heterogeneity and non-stationary elements seen in our dataset. Particularly, greater sampling over within-day/between-day physiological changes, diversity of emotion events, variation in non-emotion context, and a wider breadth of the population will lead to a better empirical understanding of the emotion-PPG relationship. With balance, broader sampling needs to be thoughtfully weighted against the accuracy and temporal granularity of emotion labelling strategies. Additionally, exploration into different physiological window sizes and stride lengths could uncover further temporal dynamics within this relationship.

Furthermore, this study took a binary view of anger when examining the anger-PPG relationship. As such, it is not sensitive to changes in intensity of emotion, differences in motivating drivers of emotion, or ambiguity in emotional states. Emotional states that were perhaps ambiguous (i.e., true subjective disagreement between real-time felt emotion and a retrospective emotion label) and not due to labelling error, were not included in this study. Future studies can aim to integrate ambiguity or uncertain in an emotion label into the modelling process.

Likewise, our characterization of overall participants was more limited to demographic attributes. With the need to better understand how PPG can be normalized across day-to-day/individual differences, further characterization of the sensor-tissue interface and anatomical differences would be useful. This includes, but is not limited to, changes in body mass, skin tone, and blood vessel sizing. Additionally, other physiological sensors may also help to characterize these differences within a modelling approach. When considering possible confounds related to TBI’s influence on ANS emotion reactivity, further experimentation could look at regional brain deficits (as characterized by functional brain imagining during emotion) and its implication to ANS response.

Lastly, this study was an initial groundwork exploration into the feasibility of anger detection within the TBI population, however we did not specifically select for individuals that had clinical presentation of severe anger dyscontrol. Future work should look to explore ANS response of these individuals during dyscontrol episodes in naturalistic settings.

## Conclusion

This study presents an initial empirical view into the characteristics of the PPG waveform and the anger-PPG (indicand-indicator) relationship as it varies through a diverse, heterogeneous dataset that includes individuals with TBI. Our dataset is a first in affective computing literature that includes this TBI population. Additionally, this dataset includes diverse endogenous/exogenous elicitation material over two days of sampling. Results highlight how PPG features have non-stationary characteristics, yet structure associated with day-to-day differences, inter-individual differences, and population sub-groups. Furthermore, there is evidence to support a contextually modulated anger-PPG relationship, in that, there are times where PPG features identifying anger changed substantially over different days of sampling, and other times where these features remained congruent. This presents a challenging problem for the creation of emotion recognition systems, as future classification models will have to be designed in a way to account for both these non-stationary and contextual changes. Without sensitivity to contextual change, PPG remains an unreliable indicator signal for anger. Future work utilizing PPG as an indicator signal to emotion should attempt to integrate contextual state variables in modeling the emotion-physiology system. We encourage future researchers to explore more heterogeneous emotion-physiology datasets for the development of these emotion recognition systems, as well as to broaden a methodological base for interrogating emotion-physiology relationships in such datasets.

## Data Availability

Not all participants consented to open sharing of their data, as such the TRIEP dataset it is not available for public use.

## Abbreviations

AMI: Auto Mutual Information
ANS: Autonomic Nervous System
AR: Autoregressive
BP: Blood pressure
BVP: Blood volume pulse
CV: Cross validation
DBP: Diastolic blood pressure
E-AI: Engebretson's anger induction
EEG: Electroencephalogram
FDR: False discovery rate
FPA: Finger pulse amplitude
FPV: Finger pulse volume
GAPED: Geneva affective picture database
GARCH: Generalized AutoRegressive Conditional Heteroskedasticity
HCTSA: Highly Comparable Time Series Analysis
HR: Heart Rate
HRV: Heart rate variability
IAPS: International affective picture system
LED: Light Emitting Diode
LMS: Least Mean Square
LOSOCV: Leave-one-session-out Cross Validation
MPCA: Multivariate Pattern Classification Analysis
PPG: Photoplethysmography
RBF: Radial Basis Function
RR: Respiration Rate
SBC: Schwartz's Bayesian Criterion
SBP: Systolic Blood Pressure
SV: Stroke Volume
TBI: Traumatic Brain Injury
T_e_: Expiration time
T_i_: Inspiration time
TPR: Total peripheral resistance
t-SNE: T-distributed Stochastic Neighbor Embedding
TRIEP: Toronto rehabilitation institute emotion-physiology dataset
UMAP: Uniform manifold approximation and projection
VMIP-E: Velten Mood Induction Procedure for Elation
